# Sodium Thiosulfate for Calciphylaxis Treatment in Patients on Peritoneal Dialysis: A Systematic Review

**DOI:** 10.3390/medicina59071306

**Published:** 2023-07-14

**Authors:** Christy Gossett, Supawadee Suppadungsuk, Pajaree Krisanapan, Supawit Tangpanithandee, Charat Thongprayoon, Michael A. Mao, Wisit Cheungpasitporn

**Affiliations:** 1 Division of Nephrology and Hypertension, Mayo Clinic, Rochester, MN 55905, USA; gossett.christy@mayo.edu (C.G.); s.suppadungsuk@hotmail.com (S.S.); pajaree_fai@hotmail.com (P.K.); supawit_d@hotmail.com (S.T.); charat.thongprayoon@gmail.com (C.T.); 2 Chakri Naruebodindra Medical Institute, Faculty of Medicine Ramathibodi Hospital, Mahidol University, Samut Prakan 10400, Thailand; 3 Division of Nephrology, Department of Internal Medicine, Faculty of Medicine Thammasat University, Pathum Thani 12120, Thailand; 4 Division of Nephrology and Hypertension, Mayo Clinic, Jacksonville, FL 32224, USA

**Keywords:** calciphylaxis, calcific uremic arteriolopathy (CUA), sodium thiosulfate (STS), peritoneal dialysis, treatment

## Abstract

Limited data are available on the utilization of sodium thiosulfate (STS) treatment for calciphylaxis in peritoneal dialysis (PD) patients, while it is well-studied in hemodialysis (HD) patients. A systematic literature search was conducted using Ovid MEDLINE, EBM Reviews—Cochrane Central Register of Controlled Trials, and EBM Reviews—Cochrane Database of Systematic Reviews to identify reported cases of PD patients with calciphylaxis who received STS. The search covered the inception of the databases through August 2022. Across 19 articles, this review identified 30 PD patients with calciphylaxis who received STS. These included 15 case reports, 2 case series, and 2 cohort studies. The administration routes and doses varied depending on the study. For intravenous (IV) administration (*n* = 18), STS doses ranged from 3.2 g twice daily to 25 g three times weekly for 5 weeks to 8 months. Outcomes included 44% of patients experiencing successful wound healing, 6% discontinuing STS due to adverse effects, 67% transitioning to HD, and 50% dying from calciphylaxis complications. For intraperitoneal (IP) administration (*n* = 5), STS doses ranged from 12.5 to 25 g three to four times weekly for 12 h to 3 months. Results showed 80% of patients achieving successful wound healing, 80% discontinuing STS due to adverse effects, 40% transitioning to HD, and 20% dying from IP STS-related chemical peritonitis. In cases where patients switched from IV to IP STS (*n* = 3), doses ranged from 12.5 to 25 g two to three times weekly for 2.5 to 5 months. Among them, 67% experienced successful wound healing, while 33% died from sepsis. Two cases utilized oral STS at a dose of 1500 mg twice daily for 6 and 11 months, resulting in successful wound healing without adverse effects or need for HD. However, one patient (50%) died due to small bowel obstruction. This systematic review provides an overview of STS treatment for PD patients with calciphylaxis. Although successful treatment cases exist, adverse effects were significant. Further research, including larger clinical studies and pharmacokinetic data, is necessary to establish the optimal route, dose, and efficacy of STS in PD patients.

## 1. Introduction

Calciphylaxis, also known as calcific uremic arteriolopathy (CUA) or uremic small-vessel disease, is an infrequent yet serious complication that can develop in patients with end-stage kidney disease (ESKD) [1,2,3]. The condition arises due to the deposition of calcium in the small blood vessels of the skin and other organs, resulting in painful tissue necrosis and skin ulcers [4,5,6]. While the exact cause of calciphylaxis remains unknown, an imbalance in calcium and phosphate metabolism is thought to contribute to its development [7,8,9,10]. Various studies have indicated that the incidence of calciphylaxis is higher in patients undergoing peritoneal dialysis (PD) compared to those receiving hemodialysis (HD) [1,11,12,13]. The incidence of calciphylaxis was reported to be 4.1–9.0 cases per 1000 patient-years in PD patients, whereas it was 0.4–3.5 cases per 1000 patient-years in HD patients [11,14,15,16,17,18]. Although the overall incidence of calciphylaxis is low in both PD and HD patients, there is a higher likelihood of its occurrence in PD patients [18].

The reasons behind the higher incidence of calciphylaxis in PD patients in comparison to HD patients are not yet fully understood [17,19]. However, several factors have been suggested to contribute to the increased risk. Firstly, PD patients are likely to have a higher exposure to calcium-containing dialysate fluids, which could contribute to the development of calciphylaxis [17]. During PD, the peritoneal membrane is used to filter blood, and it can absorb calcium from the dialysate fluid [17]. This may result in higher calcium levels in the blood, which can increase the risk of calcification in small blood vessels. Secondly, PD patients may have a higher prevalence of comorbidities such as diabetes and obesity, which are known to be risk factors for calciphylaxis [7]. Thirdly, PD patients may have a higher rate of prescription for calcium and vitamin D supplements to manage their calcium and phosphate levels [9,17,20]. This may increase the risk of calciphylaxis. Finally, PD patients may have impaired clearance of calcium and phosphate due to their reduced kidney function. This can result in an imbalance in calcium and phosphate metabolism in the body, leading to an increased risk of calciphylaxis [7,19,21].

At this stage, when addressing calciphylaxis, personalizing the dialysis treatment can provide a more individualized and tailored approach to controlling hyperparathyroidism in uremic patients. This strategy, combined with the management of the diet to include lesser-known sources of phosphorus, plays a crucial role in preventing the overload of food, tissue, and vascular fluids [22,23]. The treatment of calciphylaxis requires aggressive wound care to promote healing and prevent infection [2]. Furthermore, interventions to correct the underlying calcium and phosphate imbalance are necessary [24]. In some cases, surgical removal of the necrotic tissue and enhancing blood flow to the affected region may be essential [25]. While the exact mechanism of action of sodium thiosulfate in the treatment of calciphylaxis is not completely understood, studies suggest that sodium thiosulfate can effectively treat calciphylaxis by binding to calcium ions in the bloodstream and soft tissue, preventing the formation of calcium deposits and reducing tissue calcification [26,27,28,29,30]. Additionally, it possesses antioxidant and anti-inflammatory properties that can help reduce tissue damage and promote healing [31].

Among hemodialysis patients, typically, a 25% solution of sodium thiosulfate is administered after every hemodialysis session. The recommended dose of sodium thiosulfate is around 25 g, although this can vary depending on factors such as the patient’s weight. Treatment duration may last for several weeks or months, with dose adjustments made based on the patient’s response to the medication [32,33,34,35]. While some studies have suggested its potential benefits among PD patients for treating calciphylaxis, limited information is available and the dosing and administration of sodium thiosulfate in PD patients differ from that in HD patients [11,30,36,37,38]. Additionally, the outcomes of PD patients with calciphylaxis after sodium thiosulfate treatment are limited.

Thus, we conducted this systematic review to assess outcomes of reported cases of PD patients with calciphylaxis who received sodium thiosulfate.

## 2. Materials and Methods

### 2.1. Information Sources and Search Strategy

The researchers conducted a comprehensive literature search to identify relevant studies. The search was performed in Ovid MEDLINE, EMBASE, EBM Reviews—Cochrane Central Register of Controlled Trials, and EBM Reviews—Cochrane Database of Systematic Reviews. Medical subject headings (MeSH terms) and keywords related to calciphylaxis, peritoneal dialysis, and sodium thiosulfate were used. The search strategy aimed to retrieve all potentially relevant studies. 

## Ovid MEDLINE Search

In Ovid MEDLINE, a combination of MeSH terms and keywords were employed to identify relevant studies. The MeSH terms used were “calciphylaxis”, “peritoneal dialysis”, and “sodium thiosulfate”. Additionally, keywords were used to expand the search, including “calcific uremic arteriolopathy”, “peritoneal dialysis”, and “sodium thiosulfate”. The MeSH terms and keywords were combined using the Boolean operator “AND”.

## EMBASE Search

For EMBASE, a similar strategy was applied. Emtree terms (equivalent to MeSH terms in MEDLINE) such as “calciphylaxis”, “peritoneal dialysis”, and “sodium thiosulfate” were used. In addition, keywords similar to those used in MEDLINE, including “calcific uremic arteriolopathy”, “peritoneal dialysis”, and “sodium thiosulfate” were used. The terms were combined using the Boolean operator “AND”.

## Cochrane CENTRAL and Database of Systematic Reviews Searches

In both Cochrane CENTRAL and Cochrane Database of Systematic Reviews, similar search strategies were employed. The MeSH terms used were “calciphylaxis”, “peritoneal dialysis”, and “sodium thiosulfate”. Free text search terms were also utilized to capture any relevant studies not indexed by MeSH terms. The terms were combined with the Boolean operator “AND”.

The search was performed from the inception of the databases through August 2022. No restrictions were placed on publication date or language. The PRISMA (Preferred Reporting Items for Systematic Reviews and Meta-Analysis) [39] statement (online Supplementary Materials) guided the study’s execution. The study provides access to the data supporting its findings via the Open Science Framework (https://osf.io/2jvgf/ accessed: 7 September 2022).

### 2.2. Selection Criteria

The inclusion and exclusion criteria were pre-established to ensure the selection of relevant studies. Studies were included if they reported on PD patients with calciphylaxis who received STS treatment. Case reports, case series, and cohort studies were considered eligible. Studies that did not provide information on STS treatment or did not focus on PD patients were excluded. The screening of titles, abstracts, and full-text articles was performed independently by two reviewers. Any disagreements were resolved through discussion or consultation with a third reviewer.

### 2.3. Data Abstraction

Data extraction was carried out by trained reviewers using a standardized data abstraction form. The form captured relevant information from the included studies, including patient demographics, STS administration route and dosage, treatment outcomes, adverse effects, need for hemodialysis transition, and complications or deaths associated with calciphylaxis or STS treatment. Data abstraction was conducted independently by two reviewers, and any discrepancies were resolved through consensus or consultation with a third reviewer.

### 2.4. Evaluation of Bias Risk

Two independent reviewers performed the bias risk evaluation using various standardized tools suitable for each type of study incorporated. Any disagreements that arose were managed through discussion or by involving a third reviewer.

When analyzing case reports, the JBI Critical Appraisal Checklist for Case Reports was applied [40]. This checklist uses eight specific criteria to determine the quality of case reports, such as patient demographics clarity, diagnosis accuracy, outcome measurement appropriateness, and the impartiality of the intervention and outcome description.

The NIH Quality Assessment Tool for Case Series Studies was utilized for case series [41]. This tool scrutinizes the bias risk across nine domains, encompassing the research question’s clarity, the comprehensiveness of the case series, the uniformity of data collection, and the validity of the statistical analysis.

For cohort studies, we implemented the ROBINS-I tool [42]. This tool reviews seven bias domains: confounding elements, participant selection, intervention classification, deviations from planned interventions, missing data, outcome measurement, and result reporting. All studies underwent bias risk evaluation and received a classification of “low”, “moderate”, “serious”, or “critical” risk of bias based on the combined scores across relevant domains.

### 2.5. Statistical Analysis

Due to the heterogeneity of the included studies and the lack of raw data, a formal statistical analysis was not performed. Instead, a descriptive analysis was conducted to summarize the findings. The extracted data were presented as frequencies and percentages for categorical variables, and ranges for continuous variables, when applicable. This approach allowed for a comprehensive summary of the treatment outcomes and adverse effects reported in the included studies.

## 3. Results

**Figure 1:**
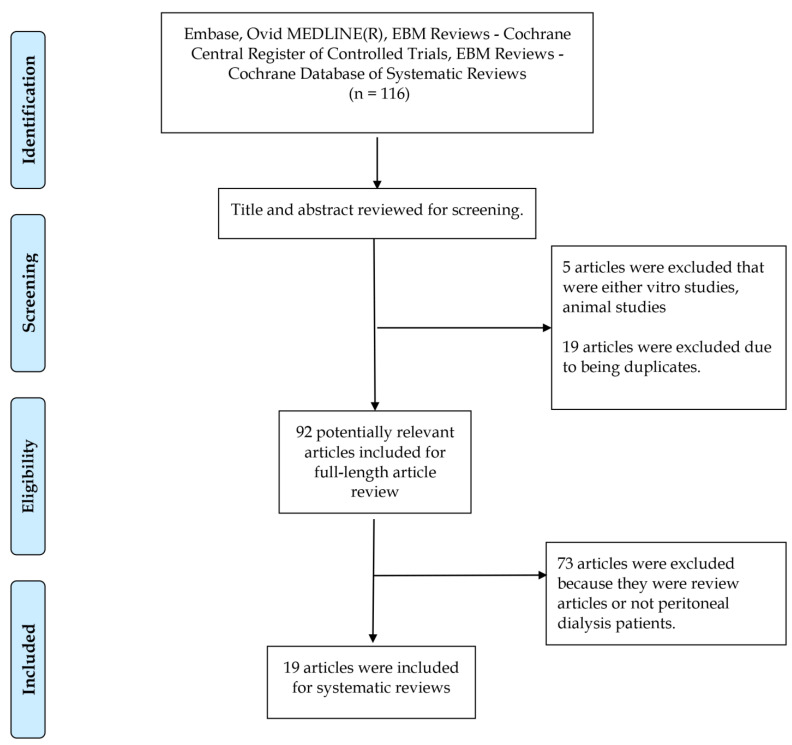
Flow diagram outlining the process of article selection and screening conducted for this research study.

The flow diagram shown in Figure 1 outlines the process of article selection and screening conducted for this research study. Initially, a total of 116 articles from Embase, Ovid MEDLINE, EBM Reviews—Cochrane Central Register of Controlled Trials, and EBM Reviews—Cochrane Database of Systematic Reviews were considered. Title and abstract screening resulted in the exclusion of five articles that were in vitro or animal studies. Additionally, 19 duplicate articles were identified and removed. Out of the remaining 92 articles, which underwent full-length article review, 73 were excluded as they were either review articles or not relevant to peritoneal dialysis patients. Finally, 19 articles met the inclusion criteria and were selected for systematic reviews.

**Figure 2:**
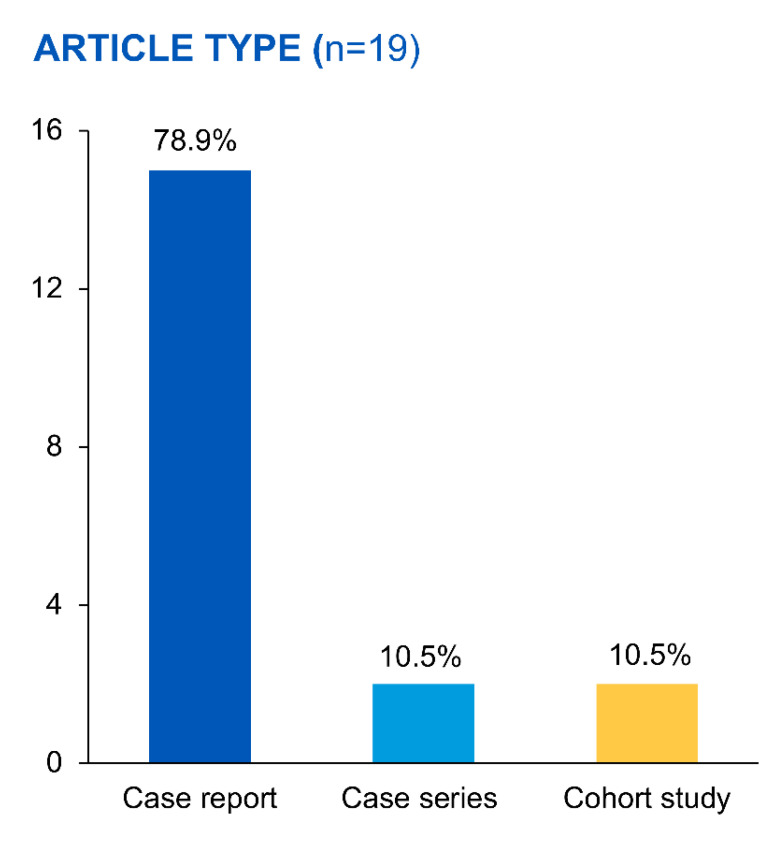
The percentage of published articles in each type of study design.

Across the 19 articles, this review identified 30 PD patients with calciphylaxis who received STS (Figure 2). 

These included 15 case reports, 2 case series, and 2 cohort studies (Table 1). The presented table offers a comprehensive overview of numerous studies and case reports investigating the treatment and outcomes of calciphylaxis, with a specific emphasis on the utilization of sodium thiosulfate. The studies, conducted over a span of nearly two decades from 2004 to 2022, provide a broad view of the varied cases of calciphylaxis, with differences in the types, locations, and severities of skin lesions. The age of the patients in the examined studies spans a broad spectrum from 17 to 85 years (Table 2), with a noteworthy female preponderance representing 63% of the total patient pool. The duration of PD administration exhibits substantial variation, ranging from as short as 3 months to as long as 10 years. Data on the adequacy of dialysis, quantified by kt/v values, are unfortunately sparse; nonetheless, where reported, these values oscillate between 1.2 and greater than 2.1. A heterogeneous patient demographic is reflected in the ethnicity/race information, encompassing a variety of racial/ethnic groups such as Caucasian, African American, Asian American, Latina, Black, White, and Chinese. Among those with a specified ethnicity/race, Caucasian patients constitute the majority, comprising approximately 50% of these particular cases. Comorbidity profiles of these patients illustrate a diverse array of health conditions, with hypertension emerging as the most prevalent. Other frequently encountered comorbidities include various cardiovascular conditions and diabetes, indicating a multifaceted health profile prevalent within this PD patient population with calciphylaxis.

Treatment durations for sodium thiosulfate, commonly administered intravenously, ranged from several weeks to multiple months. Together with sodium thiosulfate, various supplementary treatments were used, including changes in diet, the cessation of certain medications, the introduction of noncalcium binders, opioids, and specialized wound care. Additionally, surgical methods such as parathyroidectomy were employed in some cases. 

The efficacy of sodium thiosulfate treatment, as evidenced by these studies, exhibits a wide range. There were instances where patients showed notable improvement. New et al. (2011) [17] noted that the administration of sodium thiosulfate resulted in wound resolution in four out of five patients, although one patient unfortunately succumbed to sepsis. Gupta et al. (2012) [43] reported a case where the patient, despite receiving sodium thiosulfate, switched to continuous renal replacement therapy and ultimately passed away. In contrast, Mallett et al. (2012) [44] documented a successful case of sodium thiosulfate treatment for calciphylaxis, leading to lesion healing and absence of recurrence. Similarly, Torres et al. (2018) [36] discontinued sodium thiosulfate due to severe nausea, but significant pain reduction and partial wound healing were achieved through the use of low-calcium dialysate. These findings collectively underscore the variable response exhibited by patients to sodium thiosulfate and emphasize the necessity for tailored treatment approaches that consider individual patient characteristics and adherence to the prescribed regimen.

Another notable case study conducted by Danijela Mataic and Bahar Bastani (2006) [36] highlighted the initial improvement of wounds in a patient presenting multiple calciphylaxis lesions following sodium thiosulfate administration. However, recurrence of the condition and sepsis occurred due to poor compliance and the introduction of intraperitoneal (IP) sodium thiosulfate. Conversely, Dethloff (2012) [45] successfully demonstrated complete wound healing through the application of sodium thiosulfate treatment in a single case study. Furthermore, Janom et al. (2021) [46] achieved favorable outcomes by employing sodium thiosulfate in conjunction with peritoneal dialysis and subtotal parathyroidectomy for the treatment of calciphylaxis. In contrast, Zhang et al. (2016) [11] reported wound improvement with intravenous (IV) sodium thiosulfate; nevertheless, 75% of the patients eventually transitioned to hemodialysis and encountered a one-year mortality rate due to sepsis. Sood et al. (2011) [47] presented mixed results, with some patients experiencing an exacerbation of wound intensity while others displayed wound reduction; unfortunately, two patients succumbed to sepsis within one year. Finch et al. (2010) [48] documented complete wound resolution following IV sodium thiosulfate treatment. Overall, these findings underscore the inherent variability in patients’ responses to sodium thiosulfate therapy and emphasize the crucial role of individual patient characteristics and compliance with treatment protocols in achieving favorable outcomes.

**Table 1:** Characteristics of the included studies.

Author Name	Year	Type of Study	N	Location of Calciphylaxis Skin Lesions	Sodium Thiosulfate			Other Adjunctive Treatments	Dialysis Adjustment	Outcomes (Description)
					Dose	Route	Treatment Duration			
Cicone et al. [26]	2004	Case study	1	Bilateral calves and thighs	25 g 3×/week	IV	8 months (attempts at d/c earlier were met with resistance by family and patient)	Calcitriol and calcium acetate stopped, sevelamer binder, prednisone	None	Dramatic pain reduction at 2 weeks and no pain by 8 weeks, reduction in plaque size, improvement in bone scans
Danijela Mataic and Bahar Bastani [36]	2009	Case study	1	Proximal left arm and right lateral and left inner thigh	IV dose 25 g 3×/week; IP 25 g/2 L in long dwell every other day	IV initially; IP after recurrence at 25 g/2 L in long dwell every other day	2 months IV before d/c due to intolerance; 3 months of IP	low-calcium (2.5 meq/L) dialysate, wound care, parenteral antibiotics	Low calcium dialysate	Wounds improved but then recurrence due to poor compliance; IP Na thiosulfate introduced at this point— lesions progressed, sepsis and death
Amin et al. [49]	2010	Case study	1	Bilateral first metatarsals	25 g 3×/week	IV	Months	d/c calcium carbonate binder and vitamin d analogs, used noncalcium-based binders, HBO, dietary modification	Added mid-day exchange	Wound progressed and after 2 months, had to switch to HD
Finch et al. [48]	2010	Case study	1	Not listed	5 g 3/week	IV	6 months	Opioids for pain control	None	Complete resolution of wounds
New et al. [17]	2011	Observational retrospective cohort	5	Lower extremities	25 g IV (3 pts); 12.5 g IV (2 pts)	IV (3 pts) IP (3 pts)	IV- 3 mo, 6 mo, 5 weeks; IP- 3 mo	HBO, cinacalcet, parathyroidectomy, pamidronate, d/c calcium and calcitriol, change phosphate binders	3/5 patients eventually changed to HD after worsening wounds (2 pts) or 2 episodes of peritonitis (1 pt)	Resolution of wounds ×4; 1 died from sepsis Two patients who had resolution of wounds died much later from other causes (one due d/c dialysis due to functional decline; one due ischemic CCF)
Sood et al. [49]	2011	Case series	4	Lower extremities, buttocks, abdomen	25 g IV 3×/week	IV	4–14 weeks	D/c warfarin if able, d/c calcium-based binders/vit d analogs, used sevelamer, cinacalcet, IV pamidronate, antibiotics, wound care, opioids, parathyroidectomy	2 pts with increased intensity (what was done to increase is not described)	2/4 with reduced wounds (1 with complete resolution); 3/4 eventually had to switch to HD; 2/4 pts died r/t sepsis by 1 year; ¼ with reduction in pain
Dethloff, Steven B. [45]	2012	Case Study	1	Distal extremities	Initially 25 g, then decreased to 12.5 g due to nausea before transitioning to IP 25 g	IV initially but transitioned to IP due to intolerance of IV	10 weeks	Increased protein intake, phosphorus restriction, binders changed to noncalcium, calcitriol discontinued, strict BP control, pain control with hydrocodone	None described	Completely healed by 12 weeks
Gupta et al. [43]	2012	Case study	1	Medial calf (left)	25 g/2 L dialysate	IP	3 exchanges in a 12 h time frame	Calcitriol discontinued, wound care	Switched to CRRT after severe decompensation (not as part of calciphylaxis treatment plan)	Patient developed chemical peritonitis, decompensated rapidly and died days later
Mallett et al. [44]	2012	Case study	1	Distal left leg	25 g every other day ×3 doses, then 12.5 g every other day (decreased due to nausea)	IP	6 weeks	Binder changed to sevelamer, hyperbaric oxygen therapy, and wound care; aspirin; SLE was treated with mycophenolate, increase in prednisone, and hydroxychloroquine	No change	Healed lesion, biopsy 6 months later with no calciphylaxis or SLE; had successful pregnancy with post-partum SLE flare but no recurrence of calciphylaxis
Anupkumar Shetty, Jeffrey Klein [50]	2016	Case report	2	Pt 1- L middle finger, L first toe, abdominal all Pt 2-R fingers	1500 mg BID	Oral	11 mo, 6 mo	Amputation, gabapentin, opioids	None	Healed; 1 patient died of SBO 14 months later (not calciphylaxis related)
Zhang et al. [11]	2016	Cohort study—retrospective observational	4	Lower extremities, penis	25 g 3×/week	IV	2.8–5.1 months (3 m median)	Wound care/ debridement, opioids for pain, nutrition consult, surgical debridement, HBO	None	75% mortality at 1 year due to sepsis (also the same patients who eventually had to transition to HD)
Machavarapu et al. [51]	2018	Case Study	1	Esophagus	Not specified	IV	2 months	PPI, supplemental protein shakes	No change initially, transitioned to iHD eventually due to infected PD catheter	Died 2 months after presentation due to suspected spontaneous coronary event
Torres et al. [36]	2018	Case study /abstract	1	Penis	Not stated	IP	2 weeks—stopped due to severe nausea	Low calcium dialysate	Low calcium dialysate	Significant reduction in pain and some wound healing
Bara Zhaili, Khalid Al-Talib [52]	2019	Case Study	1	Right calf	4–5 mL once every 2 weeks	Intralesional	9 weeks	Wound care, PO sevelamer, IV ceftazidime, collagenase ointment	None	Complete resolution of wounds; eventually transitioned to HD due to peritonitis, not due to calciphylaxis
Tangkham et al. [53]	2019	Case study	1	Bilateral thighs (R first, then left)	12.5 mg 3×/week	IV	3 months	IV ciprofloxacin, wound care, discontinuation of calcium-containing phosphate	No changes, continued CAPD 8 h per day	Refused surgical debridement and died 3 months after presentation due to sepsis
Deng et al. [54]	2020	Case study	1	R shoulder and R fingers	6 g per day	IV	55 days	Parathyroidectomy, cinacalcet, sevelamer, antibiotics	6 days per week CAPD, 1 day per week iHD added	Amputation of 1 finger, improvement in wounds after 2 months
Di et al. [55]	2020	Case study	1	Neck, shoulders, upper extremities	6.4 g/day	Not listed	21 days	None listed	None listed	Diminished skin lesions
Janom K et al. [46]	2021	Case Study	1	Lower extremity	12.5 g in 1 L of NS as a long day dwell	IP (initially IV but severe nausea necessitated change)	3 months	Subtotal parathyroidectomy	None	Lesions healed after 6 months; mild decrease in kt/v; PD effluent cell counts monitored with no change noted
Lu et al. [30]	2022	Case study	1	Fingers and toes	3.2–6.4 g per day	IV	6 months	Calcium stopped, wound care, low calcium dialysate, lanthanum for binder, PD adjustment per Kt/V protocol	Per kt/v protocol	Healed after 9 months

Abbreviations: CAPD—continuous ambulatory peritoneal dialysis; CRRT—continuous renal replacement therapy; HBO—hyperbaric oxygen therapy; HD—hemodialysis; iHD—intermittent hemodialysis; IP—intraperitoneal; IV—intravenous; kt/v—parameter used to measure dialysis adequacy; NS—normal saline; PD—peritoneal dialysis; PPI—proton pump inhibitor; SBO—small bowel obstruction; SLE—systemic lupus erythematosus.

**Table 2:** Demographics and comorbidities.

Author	Age	Sex	PD Duration/ Type of PD	Adequacy (kt/v)	Ethnicity/Race	Cause of Renal Failure	Comorbidities
Cicone et al.	69 years	Female	3 months (CAPD)	>2.1	Caucasian	Hypertension and chronic hydronephrosis from renal calculi	Coronary artery disease, obesity, renal calculi, osteoarthritis, Graves’ disease, osteoporosis, hypertension
Danijela Mataic and Bahar Bastani	26 years	Female	4 years (CCPD)	n/a	Caucasian	Focal segmental glomerulosclerosis	n/a
Amin et al.	17 years	Male	3 years	n/a	African American	Wegner’s granulomatosis	n/a
Finch et al.	85 years	Female	n/a (CCPD)	n/a	n/a	n/a	n/a
New et al.							
#1	79 years	Female	9 years (CAPD)	n/a	n/a	Unknown	Hypertension, ischemic heart disease peripheral vascular disease, dyslipidemia, depression
#2	67 years	Male	7 months (CAPD)	n/a	n/a	Focal segmental glomerulosclerosis	Hypertension, ischemic heart disease dyslipidemia, obstructive sleep apnea, benign prostatic hyperplasia, gastroesophageal reflux disease
#3	75 years	Male	7 months (CAPD)	n/a	n/a	Diabetes, obstructive	Diabetes, ischemic heart disease, dyslipidemia, cerebrovascular accident, gastroesophageal reflux disease, hypertension, gout
#4	74 years	Male	3 years (CAPD)	n/a	n/a	Autosomal dominant polycystic kidney disease	Peripheral vascular disease, hypertension, gout, exsmoker
#5	28 years	Female	27 months (CAPD)	n/a	n/a	Systemic Lupus Erythematosus	Autoimmune hemolytic anemia, epilepsy, hypertension
Sood et al.							
#1	27 years	Female	7 years	n/a	Caucasian	Reflux nephropathy	Peripheral vascular disease, congestive heart failure, hypertension
#2	53 years	Female	6 months	n/a	Caucasian	Obstruction	Coronary artery disease, peripheral vascular disease, cerebrovascular accident, diabetes mellitus type 2
#3	63 years	Female	8 years	n/a	Caucasian	Myeloma kidney	None
#4	49 years	Female	3 years	n/a	Not Caucasian (not defined further)	Diabetes mellitus, type 2	Diabetes type 2, hypertension
Dethloff, Steven B.	56 years	Female	18 months (CCPD)	n/a	Asian American	Diabetes mellitus, type 2	n/a
Gupta et al.	82 years	Female	n/a	n/a	n/a	Lupus nephritis	Coronary artery disease, obstructive airway disease, Sjogren syndrome, obesity
Mallett et al.	30 years	Female	2 years (CAPD)	“Adequate, stable”	Caucasian	Class 4 lupus nephritis	Autoimmune hemolytic anemia, lupus anticoagulant antibody positivity without thrombosis, and seizure disorder
Anupkumar Shetty, Jeffrey Klein							
#1	55 years	Female	4 months	n/a	Latina	Diabetes	Diabetes, ovarian cancer with multiple abdominal surgeries
#2	51 years	Male	n/a	n/a	n/a	Failed kidney transplant (original cause not defined, but presumed diabetes as that is the only comorbidity listed)	Diabetes
Zhang et al.							
#1	41 years	Female	7 years	2.12	Black	Lupus	n/a
#2	34 years	Male	3.4 years	1.61	White	Lupus	n/a
#3	59 years	Female	4.1 years	1.99	Black	Unknown	n/a
#4	65 years	Female	4.8 years	2.06	White	Diabetes	n/a
Machavarapu et al.	57 years	Female	17 months	n/a	n/a	Not specified	Diabetes, hypertension, STEMI with EF 20%, triple vessel coronary artery disease
Torres et al.	63 years	Male	n/a	n/a	n/a	Not specified	Hypertension, diabetes type 2, peripheral vascular disease, poor medication compliance
Bara Zhaili, Khalid Al-Talib	51 years	Male	18 months	n/a	n/a	Diabetic nephropathy	Dilated nonischemic cardiomyopathy with diastolic dysfunction, uncontrollable hypertension, diabetic retinopathy
Tangkham et al.	43 years	Male	10 years (CAPD)	n/a	Asian	Not specified	Osteoporosis, secondary hyperparathyroidism status post subtotal parathyroidectomy, hypertension, dyslipidemia, ex-smoker, prior cannabis use
Deng et al.	33 years	Male	5 years (CAPD)	“Insufficient”	Chinese	Unknown	n/a
Di et al.	32 years	Male	~3 years (CAPD)	n/a	n/a	Not specified	Hypertension, hepatitis B
Janom K et al.	80 years	Female	n/a	“Modest, unexplained decrease in kt/v was noted”	n/a	Not specified	n/a
Lu et al.	40 years	Male	7 years	1.2	Chinese	Unknown	Hypertension, secondary hyperparathyroidism status post total parathyroidectomy with partial forearm implant 6 months prior

Abbreviations: CAPD—continuous ambulatory peritoneal dialysis; CCPD—continuous cycling peritoneal dialysis; EF—ejection fraction; kt/v—dimensionless number used in medicine to quantify hemodialysis and peritoneal dialysis treatment adequacy; n/a—not available or not applicable; PD—peritoneal dialysis; STEMI—ST-elevation myocardial infarction. References with multiple cases have each individual case and associated demographics identified by # in Table 2.

**Figure 3:**
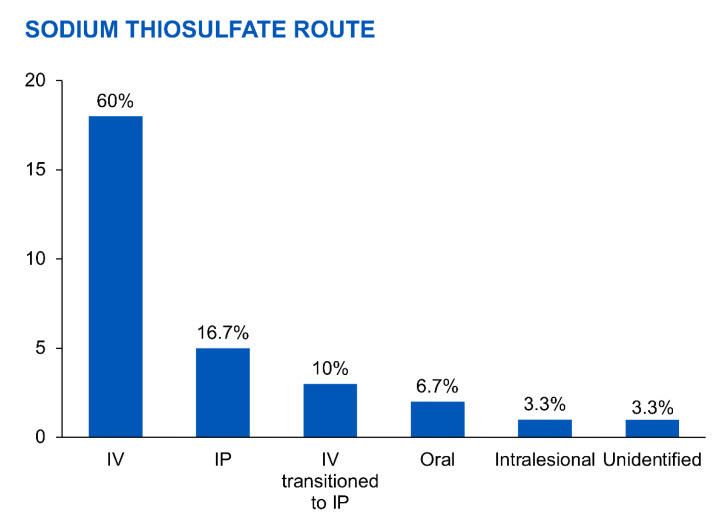
The percentage of sodium thiosulfate administration routes.

The administration routes and doses varied depending on the study (Figure 3). For intravenous (IV) administration (n = 18), STS doses ranged from 3.2 g twice daily to 25 g three times weekly for 5 weeks to 8 months. Outcomes included 44% of patients experiencing successful wound healing, 6% discontinuing STS due to adverse effects, 67% transitioning to HD, and 50% dying from calciphylaxis complications.

For intraperitoneal (IP) administration (*n* = 5), STS doses ranged from 12.5 to 25 g three to four times weekly for 12 h to 3 months. Results showed 80% of patients achieving successful wound healing, 80% discontinuing STS due to adverse effects, 40% transitioning to HD, and 20% dying from IP STS-related chemical peritonitis.

In cases where patients switched from IV to IP STS (*n* = 3), doses ranged from 12.5 to 25 g two to three times weekly for 2.5 to 5 months. Among them, 67% experienced successful wound healing, while 33% died from sepsis.

Two cases utilized oral STS at a dose of 1500 mg twice daily for 6 and 11 months, resulting in successful wound healing without adverse effects or the need for HD. However, one patient (50%) died due to small bowel obstruction.

**Figure 4:**
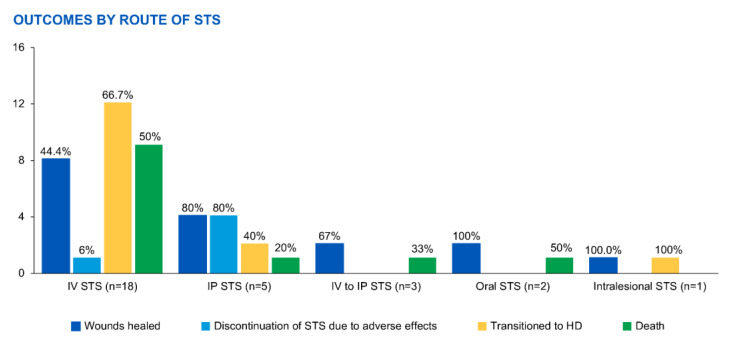
Comparison of the effectiveness of sodium thiosulfate therapy and administration routes. HD—hemodialysis; IP—intraperitoneal; IV—intravenous; STS—sodium thiosulfate.

The outcomes varied depending on the route of STS administration (Figure 4), with both successful wound healing and adverse effects observed across the different routes. The highest success rates were seen with IP administration, while the highest mortality rates were observed with IV administration and IP STS-related chemical peritonitis.

### Risk of Bias Assessment

To evaluate bias in the case reports, the JBI Critical Appraisal Checklist for Case Reports was employed [40]. This tool scrutinizes various aspects of the study, such as the appropriateness of the study design, the clarity of the research objectives and questions, the adequacy of data collection methods, the consideration of ethical issues, the transparency of data analysis, and the validity of the conclusions drawn. The assessment identified one case report [26] with a high risk of bias, indicating potential limitations in the study design, data collection, or analysis that may affect the reliability of the findings. Another case report [36] demonstrated a moderate risk of bias, implying some shortcomings but not to the extent of the high-risk study. The remaining case reports were deemed to have a low risk of bias, indicating a higher level of methodological rigor.

For the case series studies, the NIH Quality Assessment Tool for Case Series Studies was employed [41]. This tool evaluates various aspects of study design, data collection, and analysis, including the clarity of the case series objectives, the appropriateness of case selection and data sources, the completeness of data collection, the consideration of confounding factors, and the reporting of outcomes. Among the case series studies, one study [17] exhibited a moderate risk of bias, suggesting potential limitations in the study design or analysis that may impact the validity of the results. On the other hand, the other case series study [47] demonstrated a low risk of bias, indicating a higher level of methodological rigor and fewer potential sources of bias.

The risk bias assessment for the cohort studies utilized the ROBINS-I tool [43], which examines the risk of bias across several domains, including confounding, participant selection, intervention classification, deviations from intended interventions, missing data, outcome measurement, and selection of reported results. Among the cohort studies, one study [11] displayed a moderate risk of bias, suggesting potential limitations that may impact the internal validity of the study. However, none of the included cohort studies were reported to have a high risk of bias, indicating a relatively stronger methodological quality in terms of minimizing potential biases.

Overall, the risk bias assessments provide valuable insights into the methodological quality and potential biases present in the included studies. They emphasize the importance of interpreting the study findings cautiously, taking into account the limitations introduced by the identified risks of bias. These assessments underscore the significance of critically appraising the included studies to evaluate their methodological rigor and potential sources of bias. Moreover, they highlight the need for further high-quality studies with robust methodologies to strengthen the evidence base regarding the treatment and outcomes of calciphylaxis and the utilization of sodium thiosulfate.

## 4. Discussion

Calciphylaxis, a rare and severe condition characterized by the calcification and ischemic necrosis of small-to-medium-sized blood vessels in the skin and subcutaneous tissues, primarily affects ESKD patients undergoing dialysis, particularly those on PD [13,17,18,20]. STS has been utilized as a treatment option for calciphylaxis; however, its effectiveness and usage in PD patients have been less studied compared to patients undergoing HD. This systematic review aimed to explore the existing literature on the use of STS in PD patients with calciphylaxis.

The review identified 30 PD patients from 19 articles who received STS for treating calciphylaxis. The administration routes and doses of STS varied across the studies, indicating the absence of standardized protocols for PD patients. The most commonly reported method was IV administration, with doses ranging from 3.2 g twice daily to 25 g three times weekly for durations of 5 weeks to 8 months [17,56,57]. IP administration was used in a smaller subset of patients, with doses ranging from 12.5 to 25 g three to four times weekly for 12 h to 3 months [17]. In a few cases, patients switched from IV to IP administration, and oral STS was used in two instances.

The outcomes of STS treatment varied, with some patients experiencing successful wound healing, while others had to discontinue treatment due to adverse effects or experienced more severe complications. For IV administration, 44% of patients achieved successful wound healing, but 6% had to discontinue STS due to adverse effects. Additionally, 67% of patients transitioned to HD, and 50% of patients died from calciphylaxis-related complications. The outcomes were somewhat different for IP administration, with 80% of patients achieving successful wound healing but 80% discontinuing STS due to adverse effects. Among the patients who switched from IV to IP administration, 67% experienced successful wound healing, but 33% died from sepsis. The two cases involving oral STS demonstrated successful wound healing without adverse effects, but one patient died due to small bowel obstruction.

These findings emphasize the potential benefits of STS treatment in PD patients with calciphylaxis, particularly regarding wound healing. However, it is important to note that adverse effects were significant, leading to treatment discontinuation in a considerable number of cases. The adverse effects associated with STS use, such as chemical peritonitis with IP administration and sepsis in patients switching from IV to IP administration, underscore the necessity for careful monitoring and personalized dosing regimens [38,43,58,59]. The high mortality rate observed in this review also highlights the severity and complexity of calciphylaxis in PD patients [2,11,17,60].

Moreover, transitioning from PD to HD has been commonly recommended for better management of hyperphosphatemia and hyperparathyroidism, which are key factors contributing to the development and progression of calciphylaxis [61,62]. HD allows more efficient removal of phosphate and improved control of mineral and bone disorders. Consequently, the administration of STS during hemodialysis sessions has become a common practice in treating calciphylaxis [63]. This approach capitalizes on the dialysis session to deliver STS directly into the bloodstream, potentially enhancing its therapeutic effectiveness. While the reviewed studies primarily focused on STS administration in PD patients, a significant proportion of cases involved transitioning to HD [11,17]. This suggests that clinicians switch to HD to optimize calciphylaxis management and enhance patient outcomes. The transition to HD allows for more precise control of STS dosing and better monitoring of treatment response. Furthermore, HD offers the advantage of regular s-essions with close clinical supervision, facilitating the identification and management of potential adverse effects associated with STS therapy. The use of STS during hemodialysis provides a targeted treatment approach by infusing it directly into the bloodstream, enabling precise dosing and reducing the risk of complications associated with IP administration. Close monitoring during HD sessions allows for prompt identification and management of adverse effects, potentially improving the safety profile of STS therapy. Overall, transitioning from PD to HD, coupled with STS administration during hemodialysis, appears to be a prevalent strategy in calciphylaxis management. This integrated approach addresses the underlying pathophysiology, including the control of mineral and bone disorders, while also leveraging the benefits of STS therapy. However, it is crucial to consider individual patient factors such as comorbidities, vascular access, and dialysis adequacy when making treatment decisions and determining the most appropriate dialysis modality and STS administration route.

Nevertheless, it is important to acknowledge that transitioning from PD to HD may not always be feasible or recommended. Some PD patients with calciphylaxis may have limitations that prevent them from switching to HD. Hemodynamic instability can be a significant concern in certain patients, making them unsuitable candidates for hemodialysis due to underlying cardiovascular conditions or compromised hemodynamic stability. Additionally, challenging vascular access can impede the transition to HD, as some patients may have exhausted their options for vascular access due to repeated failures or complications. Limited or compromised vascular access can make hemodialysis difficult or even impossible, necessitating the continuation of PD as the primary dialysis modality. Patient preferences and autonomy also play a crucial role in treatment decisions. Despite understanding the risks and benefits associated with transitioning to hemodialysis, some patients may refuse to switch due to personal reasons, fear of change, or lifestyle considerations. In such cases, healthcare providers should respect patient autonomy and collaborate to explore alternative treatment options and optimize PD care for effective calciphylaxis management.

In clinical practice, healthcare professionals often encounter situations where calciphylaxis patients cannot be transitioned to hemodialysis due to hemodynamic instability, challenging vascular access, or patient refusal. These cases present unique challenges, requiring tailored treatment strategies to address individual patient needs and circumstances. Alternative approaches, including optimizing PD techniques, adjunctive therapies, wound care management, and supportive measures, may be employed to meet the specific requirements of these patients and improve their outcomes while on PD.

The limitations of this review include the scarcity of data on STS treatment specifically in PD patients with calciphylaxis and the heterogeneity of the included studies in terms of study design, sample size, and dosing regimens. The absence of standardized protocols and the retrospective nature of most studies hindered definitive conclusions regarding the optimal use of STS in this patient population. In order to address the existing limitations in the current literature and enhance the management of calciphylaxis, future studies in this field should focus on a range of areas. It is crucial to conduct well-designed prospective studies that can evaluate the effectiveness and safety of STS in patients undergoing PD, with particular attention to optimizing the timing, dosage, and duration of treatment. Furthermore, comparative investigations that directly compare PD and HD patients would provide valuable insights for treatment decision-making. Exploring alternative treatment modalities, such as calcimimetics, intravenous administration of tissue plasminogen activation,, and hyperbaric oxygen therapy, is also warranted. Efforts should be made to develop strategies that can mitigate adverse effects, establish standardized protocols, and evaluate long-term outcomes and cost-effectiveness. Additionally, the identification of biomarkers and imaging techniques for early diagnosis and monitoring would significantly contribute to the advancement of calciphylaxis management. By undertaking research in these areas, future studies will facilitate the development of evidence-based strategies for the effective care of patients affected by calciphylaxis.

## 5. Conclusions

This systematic review provides an overview of the utilization of STS in PD patients with calciphylaxis. While some patients achieved successful wound healing with STS treatment, adverse effects were significant, and mortality rates were high. Further research, including well-designed prospective studies, is necessary to establish standardized protocols, determine optimal dosing regimens, and assess the long-term efficacy and safety of STS in PD patients with calciphylaxis. Additionally, comparative investigations on the outcomes of STS treatment in PD and HD patients would be valuable for informing clinical decision-making and improving the management of this challenging condition.

## Data Availability

Data supporting this study are available in the original publication, reports, and preprints that were cited in the reference citation.

## Supplementary Materials

The following supporting information can be downloaded at: https://www.mdpi.com/article/10.3390/medicina59071306/s1. Reference [64] is cited in the Supplementary Materials.

## References

[1] Nigwekar S.U., Thadhani R., Brandenburg V.M. (2018). Calciphylaxis. N. Engl. J. Med..

[2] Gallo Marin B., Aghagoli G., Hu S.L., Massoud C.M., Robinson-Bostom L. (2023). Calciphylaxis and Kidney Disease: A Review. Am. J. Kidney Dis..

[3] Krisanapan P., Pattharanitima P., Thongprayoon C., Cheungpasitporn W. (2022). Recent Advances in Understanding of Cardiovascular Diseases in Patients with Chronic Kidney Disease. J. Clin. Med..

[4] Disthabanchong S., Srisuwarn P. (2019). Mechanisms of Vascular Calcification in Kidney Disease. Adv. Chronic. Kidney Dis..

[5] Fine A., Fleming S., Leslie W. (1995). Calciphylaxis presenting with calf pain and plaques in four continuous ambulatory peritoneal dialysis patients and in one predialysis patient. Am. J. Kidney Dis..

[6] Thongprayoon C., Cheungpasitporn W., Bruminhent J. (2015). Aggressive calciphylaxis in end-stage renal disease after a failed kidney allograft. Indian J. Dermatol. Venereol. Leprol..

[7] Nigwekar S.U., Kroshinsky D., Nazarian R.M., Goverman J., Malhotra R., Jackson V.A., Kamdar M.M., Steele D.J., Thadhani R.I. (2015). Calciphylaxis: Risk factors, diagnosis, and treatment. Am. J. Kidney Dis..

[8] Rogers N.M., Teubner D., Coates P. (2007). VASCULAR CALCIFICATION IN PATIENTS WITH KIDNEY DISEASE: Calcific uremic arteriolopathy: Advances in pathogenesis and treatment. Semin. Dial..

[9] Fine A., Zacharias J. (2002). Calciphylaxis is usually non-ulcerating: Risk factors, outcome and therapy. Kidney Int..

[10] Thongprayoon C., Cheungpasitporn W., Mao M.A., Erickson S.B. (2020). Calcium-phosphate product and its impact on mortality in hospitalized patients. Nephrology.

[11] Zhang Y., Corapi K.M., Luongo M., Thadhani R., Nigwekar S.U. (2016). Calciphylaxis in peritoneal dialysis patients: A single center cohort study. Int. J. Nephrol. Renovasc. Dis..

[12] Sprague S.M. (2014). Painful skin ulcers in a hemodialysis patient. Clin. J. Am. Soc. Nephrol..

[13] Fine A., Fontaine B. (2008). Calciphylaxis: The beginning of the end?. Perit. Dial. Int..

[14] Nigwekar S.U., Zhao S., Wenger J., Hymes J.L., Maddux F.W., Thadhani R.I., Chan K.E. (2016). A nationally representative study of calcific uremic arteriolopathy risk factors. J. Am. Soc. Nephrol..

[15] Angelis M., Wong L.L., Myers S.A., Wong L.M. (1997). Calciphylaxis in patients on hemodialysis: A prevalence study. Surgery.

[16] Nigwekar S.U., Solid C.A., Ankers E., Malhotra R., Eggert W., Turchin A., Thadhani R.I., Herzog C.A. (2014). Quantifying a rare disease in administrative data: The example of calciphylaxis. J. Gen. Intern. Med..

[17] New N., Mohandas J., John G.T., Ratanjee S., Healy H., Francis L., Ranganathan D. (2011). Calcific uremic arteriolopathy in peritoneal dialysis populations. Int. J. Nephrol..

[18] Brandenburg V.M., Kramann R., Rothe H., Kaesler N., Korbiel J., Specht P., Schmitz S., Krüger T., Floege J., Ketteler M. (2016). Calcific uraemic arteriolopathy (calciphylaxis): Data from a large nationwide registry. Nephrol. Dial. Transplant..

[19] Brandenburg V.M., Cozzolino M., Ketteler M. (2011). Calciphylaxis: A still unmet challenge. J. Nephrol..

[20] Harris C., Kiaii M., Lau W., Farah M. (2018). Multi-intervention management of calcific uremic arteriolopathy in 24 patients. Clin. Kidney J..

[21] Weenig R.H., Sewell L.D., Davis M.D., McCarthy J.T., Pittelkow M.R. (2007). Calciphylaxis: Natural history, risk factor analysis, and outcome. J. Am. Acad. Dermatol..

[22] Monardo P., Lacquaniti A., Campo S., Bucca M., Casuscelli di Tocco T., Rovito S., Ragusa A., Santoro A. (2021). Updates on hemodialysis techniques with a common denominator: The personalization of the dialytic therapy. Semin. Dial..

[23] Savica V., Calò L.A., Monardo P., Santoro D., Mallamace A., Muraca U., Bellinghieri G. (2009). Salivary phosphorus and phosphate content of beverages: Implications for the treatment of uremic hyperphosphatemia. J. Ren. Nutr..

[24] Udomkarnjananun S., Kongnatthasate K., Praditpornsilpa K., Eiam-Ong S., Jaber B.L., Susantitaphong P. (2019). Treatment of Calciphylaxis in CKD: A Systematic Review and Meta-analysis. Kidney Int. Rep..

[25] McCarthy J.T., El-Azhary R.A., Patzelt M.T., Weaver A.L., Albright R.C., Bridges A.D., Claus P.L., Davis M.D., Dillon J.J., El-Zoghby Z.M. (2016). Survival, Risk Factors, and Effect of Treatment in 101 Patients with Calciphylaxis. Mayo Clin. Proc..

[26] Cicone J.S., Petronis J.B., Embert C.D., Spector D.A. (2004). Successful treatment of calciphylaxis with intravenous sodium thiosulfate. Am. J. Kidney Dis..

[27] Araya C.E., Fennell R.S., Neiberger R.E., Dharnidharka V.R. (2006). Sodium thiosulfate treatment for calcific uremic arteriolopathy in children and young adults. Clin. J. Am. Soc. Nephrol..

[28] Ackermann F., Levy A., Daugas E., Schartz N., Riaux A., Derancourt C., Urena P., Lebbé C. (2007). Sodium thiosulfate as first-line treatment for calciphylaxis. Arch. Dermatol..

[29] Schlieper G., Brandenburg V., Ketteler M., Floege J. (2009). Sodium thiosulfate in the treatment of calcific uremic arteriolopathy. Nat. Rev. Nephrol..

[30] Lu Y., Shen L., Zhou L., Xu D. (2022). Success of small-dose fractionated sodium thiosulfate in the treatment of calciphylaxis in a peritoneal dialysis patient. BMC Nephrol..

[31] An J., Devaney B., Ooi K.Y., Ford S., Frawley G., Menahem S. (2015). Hyperbaric oxygen in the treatment of calciphylaxis: A case series and literature review. Nephrology.

[32] Wen W., Portales-Castillo I., Seethapathy R., Krinsky S., Kroshinsky D., Kalim S., Goverman J., Nazarian R.M., Chitalia V., Malhotra R. (2023). Intravenous sodium thiosulphate for vascular calcification of hemodialysis patients-a systematic review and meta-analysis. Nephrol. Dial. Transpl..

[33] Singh R.P., Derendorf H., Ross E.A. (2011). Simulation-Based Sodium Thiosulfate Dosing Strategies for the Treatment of Calciphylaxis. Clin. J. Am. Soc. Nephrol..

[34] Generali J.A., Cada D.J. (2015). Sodium Thiosulfate: Calciphylaxis. Hosp. Pharm..

[35] Galassi A., Perna F., De Nicola E., Moneghini L., Sganzaroli A.B., Cozzolino M. (2018). Calciphylaxis in a dialysis patient treated by intralesional and systemic sodium thiosulphate on top of multifactorial intervention. Clin. Kidney J..

[36] Mataic D., Bastani B. (2006). Intraperitoneal sodium thiosulfate for the treatment of calciphylaxis. Ren. Fail..

[37] Teh Y.K., Renaud C.J. (2023). Clinical experience with intraperitoneal sodium thiosulphate for calciphylaxis in peritoneal dialysis: A case series. Perit. Dial. Int..

[38] Roy S., Reddy S.N., Garcha A.S., Vantipalli P., Patel S.S., Ur Rahman E., Adapa S. (2021). Successful Treatment of Calciphylaxis in a Young Female With End-Stage Renal Disease on Peritoneal Dialysis With Parathyroidectomy, Intensification of Dialysis, and Sodium Thiosulphate-A Case Report and Literature Review. J. Investig. Med. High Impact Case Rep..

[39] Moher D., Liberati A., Tetzlaff J., Altman D.G. (2009). Preferred reporting items for systematic reviews and meta-analyses: The PRISMA statement. PLoS Med..

[40] Munn Z., Barker T.H., Moola S., Tufanaru C., Stern C., McArthur A., Stephenson M., Aromataris E. (2020). Methodological quality of case series studies: An introduction to the JBI critical appraisal tool. JBI Evid. Synth..

[41] National Institutes of Health (2018). National Heart, Lung, and Blood Institute. Study Quality Assessment Tool for Case Series Studies. Bethesda..

[42] Sterne J.A., Hernán M.A., Reeves B.C., Savović J., Berkman N.D., Viswanathan M., Henry D., Altman D.G., Ansari M.T., Boutron I. (2016). ROBINS-I: A tool for assessing risk of bias in non-randomised studies of interventions. BMJ.

[43] Gupta D.R., Sangha H., Khanna R. (2012). Chemical peritonitis after intraperitoneal sodium thiosulfate. Perit. Dial. Int..

[44] Mallett A., John G., Ranganathan D., Kark A., Berquier I., Casey J., Healy H., Francis L. (2012). Sustained remission of systemic lupus erythematosus related calciphylaxis. Lupus.

[45] Dethloff S.B. (2012). Calcific uremic arteriolopathy: Treatment with intraperitoneal sodium thiosulfate in a patient on peritoneal dialysis. Nephrol. Nurs. J..

[46] Janom K., Shaikhouni S., Perlman R., Swartz R.D. Revisiting Route of Therapy for Calciphylaxis in Peritoneal Dialysis. Proceedings of the Kidney Week Annual Meeting.

[47] Sood A.R., Wazny L.D., Raymond C.B., Leung K., Komenda P., Reslerova M., Verrelli M., Rigatto C., Sood M.M. (2011). Sodium thiosulfate-based treatment in calcific uremic arteriolopathy: A consecutive case series. Clin. Nephrol..

[48] Finch S., Aspden I., Johnson L., Bashir K. (2010). The Use of Intravenous Sodium Thiosulfate for the Treatment of Calciphylaxis in an Elderly Peritoneal Dialyisis Patient. J. Ren. Nutr..

[49] Amin N., Gonzalez E., Lieber M., Salusky I.B., Zaritsky J.J. (2010). Successful treatment of calcific uremic arteriolopathy in a pediatric dialysis patient. Pediatr. Nephrol..

[50] Shetty A., Klein J. (2016). Treatment of Calciphylaxis: A Case for Oral Sodium Thiosulfate. Adv. Perit. Dial..

[51] Machavarapu A., Brown T.A., Nwakoby I.E. (2018). Rare Case of Hematemesis: Calciphylaxis of the Esophagus. Clin. Gastroenterol. Hepatol..

[52] Zuhaili B., Al-Talib K. (2019). Successful Treatment of Single Infected Calciphylaxis Lesion With Intralesional Injection of Sodium Thiosulfate at High Concentration. Wounds A Compend. Clin. Res. Pract..

[53] Tangkham R., Sangmala S., Aiempanakit K., Chiratikarnwong K., Auepemkiate S. (2019). Calciphylaxis mimicking ecthyma gangrenosum. IDCases.

[54] Deng Y., Shu Y., Gong R. (2020). Calciphylaxis in patient with peritoneal dialysis: A case report. Cogent Med..

[55] Di J., Liu Y., Wang D., Yang M. (2020). A Case of Early Calciphylaxis Diagnosed by Bone Scan. Case Rep. Med..

[56] Vedvyas C., Winterfield L.S., Vleugels R.A. (2012). Calciphylaxis: A systematic review of existing and emerging therapies. J. Am. Acad. Dermatol..

[57] Hayden M.R., Goldsmith D., Sowers J.R., Khanna R. (2008). Calciphylaxis: Calcific uremic arteriolopathy and the emerging role of sodium thiosulfate. Int. Urol. Nephrol..

[58] Sherman C. (2013). Chemical peritonitis after intraperitoneal sodium thiosulfate. Perit. Dial. Int..

[59] Raymond C.B., Wazny L.D. (2008). Sodium thiosulfate, bisphosphonates, and cinacalcet for treatment of calciphylaxis. Am. J. Health Syst. Pharm..

[60] Lal G., Nowell A.G., Liao J., Sugg S.L., Weigel R.J., Howe J.R. (2009). Determinants of survival in patients with calciphylaxis: A multivariate analysis. Surgery.

[61] Kidney Disease: Improving Global Outcomes (KDIGO) CKD-MBD Update Work Group (2017). KDIGO 2017 Clinical Practice Guideline Update for the Diagnosis, Evaluation, Prevention, and Treatment of Chronic Kidney Disease-Mineral and Bone Disorder (CKD-MBD). Kidney Int. Suppl..

[62] Fernández-Martín J.L., Martínez-Camblor P., Dionisi M.P., Floege J., Ketteler M., London G., Locatelli F., Gorriz J.L., Rutkowski B., Ferreira A. (2015). Improvement of mineral and bone metabolism markers is associated with better survival in haemodialysis patients: The COSMOS study. Nephrol. Dial. Transplant..

[63] Farese S., Stauffer E., Kalicki R., Hildebrandt T., Frey B.M., Frey F.J., Uehlinger D.E., Pasch A. (2011). Sodium thiosulfate pharmacokinetics in hemodialysis patients and healthy volunteers. Clin. J. Am. Soc. Nephrol..

[64] Page M.J., McKenzie J.E., Bossuyt P.M., Boutron I., Hoffmann T.C., Mulrow C.D., Shamseer L., Tetzlaff J.M., Akl E.A., Brennan S.E. (2021). The PRISMA 2020 statement: An updated guideline for reporting systematic reviews. BMJ.

